# Effects of flavoring compounds used in electronic cigarette refill liquids on endothelial and vascular function

**DOI:** 10.1371/journal.pone.0222152

**Published:** 2019-09-09

**Authors:** Gerald Wölkart, Alexander Kollau, Heike Stessel, Michael Russwurm, Doris Koesling, Astrid Schrammel, Kurt Schmidt, Bernd Mayer

**Affiliations:** 1 Department of Pharmacology and Toxicology, Institute of Pharmaceutical Sciences, Karl-Franzens-Universität Graz, Graz, Austria; 2 Department of Pharmacology and Toxicology, Ruhr-Universität Bochum, Bochum, Germany; Max Delbruck Centrum fur Molekulare Medizin Berlin Buch, GERMANY

## Abstract

Electronic cigarette refill liquids are commercially provided with a wide variety of flavoring agents. A recent study suggested that several common flavors may scavenge nitric oxide (NO) and cause endothelial dysfunction. It was the aim of the present study to investigate the effects of these flavors on NO/cyclic GMP-mediated signaling and vascular relaxation. We tested the flavoring agents for effects on Ca^2+^-induced cGMP accumulation and NO synthase activation in cultured endothelial cells. NO scavenging was studied with NO-activated soluble guanylate cyclase and as NO release from a NO donor, measured with a NO electrode. Blood vessel function was studied with precontracted rat aortic rings in the absence and presence of acetylcholine or a NO donor. Cinnamaldehyde inhibited Ca^2+^-stimulated endothelial cGMP accumulation and NO synthase activation at ≥0.3 mM. Cinnamaldehyde and diacetyl inhibited NO-activated soluble guanylate cyclase with IC_50_ values of 0.56 (0.54–0.58) and 0.29 (0.24–0.36) mM, respectively, and caused moderate NO scavenging at 1 mM that was not mediated by superoxide anions. The other compounds did not scavenge NO at 1 mM. None of the flavorings interfered with acetylcholine-induced vascular relaxation, but they caused relaxation of pre-contracted aortas. The most potent compounds were eugenol and cinnamaldehyde with EC_50_ values of ~0.5 mM. Since the flavors did not affect endothelium-dependent vascular relaxation, NO scavenging by cinnamaldehyde and diacetyl does not result in impaired blood vessel function. Although not studied *in vivo*, the low potency of the compounds renders it unlikely that the observed effects are relevant to humans inhaling flavored vapor from electronic cigarettes.

## Introduction

Electronic cigarettes (e-cigs) vaporize liquids, typically containing propylene glycol, glycerol, nicotine, and flavoring compounds, at a temperature of 200–250°C. Current and former smokers inhale the resulting vapor instead of tobacco smoke [1]. The combustion of tobacco leaves and cigarette paper at >800°C leads to the emission of several thousand compounds, many of which are cancerogenic or otherwise toxic. According to the WHO, about 6 million smokers die each year from tobacco-associated diseases, and cigarettes kill every second user. Since e-cigs do not burn tobacco, vaping is not associated with the inhalation of combustion products. The analytical studies currently available show that at normal usage of the products the harmful constituents of tobacco smoke are either absent or present only in trace amounts in the inhaled aerosol [2, 3] and in the air of closed rooms after vaping [4, 5]. Some researchers described e-cig vapor as being similarly harmful as tobacco smoke because of the emission of particulate matter without considering the difference between the harmful solid particles in smoke and benign liquid droplets in e-cig aerosols [6]. The switch from smoking to vaping was shown to result in the pronounced reduction of biomarkers for toxicity and improvement of several clinical parameters [7, 8], demonstrating the reduction of the health risks of consumers. Clinical studies showing significant improvement of lung function of smokers with asthma [9] as well as reduced symptom load of COPD patients [10], who used e-cigs instead of combustible tobacco cigarettes, further support this view.

Despite the apparent potential of e-cigs in tobacco harm reduction, some health professionals are still reluctant to recommend these products to smokers because of several caveats. According to a report by Stanton Glantz and co-workers, e-cigs do not aid in smoking cessation but, in contrast, decrease the rate of successful quit attempts [11]. This claim contradicts a recent randomized clinical trial [12] and observational studies [13, 14], indicating that e-cigs are superior over approved nicotine replacement therapy. Another objection is the continued consumption of nicotine, which could cause adverse cardiovascular effects due to sympathetic stimulation and support the dependence of users. However, nicotine is not responsible for the high morbidity and mortality of smoking and does not increase the risk for severe cardiovascular events [15]. Observation of never smokers vaping for 3.5 years provided no evidence for impaired lung or cardiovascular function [16]. Concerning dependence, studies with rodents show that non-nicotine ingredients of tobacco smoke, e.g. inhibitors of monoamine oxidase and acetaldehyde, significantly enhance the positive reinforcing effects of nicotine [17]. In humans, the addictive potential of e-cigs appears to be similar to that of nicotine-containing chewing gums approved for therapeutic smoking cessation [18]. In addition to the substance-induced release of dopamine, airway sensation and conditioned smoking behavior significantly contribute to the dependence of smokers [19]. Vaping allows consumers to maintain their conditioned behavior and provides the throat hit necessary for satisfaction. This may explain the greater appeal and success rates of e-cigs as compared to nicotine replacement therapy [20].

Published surveys show that availability of a wide array of flavors is essential for the satisfaction of consumers and that the majority of vapers prefer fruit and other non-tobacco flavors [21, 22]. It is beyond the scope of this article to discuss the current controversy about the alleged nicotine epidemic caused by flavored e-cigs among youth, and we wish to focus on the potential health risks of inhaled flavors. Virtually all of the compounds used in refill liquids are approved as food flavorings and widely used without evidence for adverse effects. However, it cannot be excluded that some of these compounds are harmful if inhaled. For most flavors, this assumption is not supported by evidence, but there are two notable exceptions. Diacetyl, used in certain sweet liquids, was proposed to cause *Bronchiolitis obliterans* ("popcorn lung"), due to damage to lung epithelial cells [23]. Although the diacetyl concentrations in e-cig vapor are probably below occupational limits, the addition of diacetyl to e-liquids was banned in some countries, including Germany and Austria. For safety reasons, consumers are advised to refrain from using diacetyl containing liquids. The second suspicious compound is cinnamaldehyde, which was shown to be toxic to some types of cultured cells, including lung epithelial cells [24]. Whether the results of *in vitro* experiments with cultured cells exposed to e-cig vapor can be translated to the *in vivo* situation of human consumers remains to be established.

Potential toxic effects of flavors on alveolar epithelial cells may occur in the absence of significant levels in the systemic circulation, but this does not apply to effects on vascular and endothelial function. In a recent study [25], it has been claimed that certain flavors may cause endothelial dysfunction due to scavenging of nitric oxide (NO). Therefore, we tested the effects of the flavoring agents used by Fetterman et al. [25] on endothelial NO signaling, NO scavenging and NO-induced relaxation of rat aortas.

## Materials and methods

### Materials

L-[2,3,4,5-^3^H]arginine (57 Ci/mmol) and [α-^32^P]GTP (800 Ci/mmol) were from Perkin Elmer Life and Analytical Sciences (Vienna, Austria), 2,2-Diethyl-1-nitroso-oxyhydrazine (DEA/NO) was from Enzo Life Sciences (Lausen, Switzerland) obtained via Eubio (Vienna, Austria). All other chemicals, including the flavorings listed in **Table 1**, were from Sigma-Aldrich (Vienna, Austria). Stock solutions of flavorings (1 M) were prepared in DMSO and diluted as required with deionized water. The following cinnamaldehyde containing liquids were obtained from happy liquid (Munich, Germany): Auszogne mit Zimt, Glühwein, and Apfelstrudel.

**Table 1:** Flavorings tested in this study (in alphabetical order).

Compound	IUPAC Name	CAS Number
Acetylpyridine	1-(Pyridin-2-yl)ethan-1-one	1122-62-9
Cinnamaldehyde	(2*E*)-3-Phenylprop-2-enal	14371-10-9
Diacetyl	Butane-2,3-dione	431-03-8
Dimethylpyrazine	2,5-Dimethylpyrazine	1122-62-9
Eucalyptol	1,3,3-Trimethyl-2-oxabicyclo[2.2.2]octane	470-82-6
Eugenol	2-Methoxy-4-(prop-2-en-1-yl)phenol	97-53-0
Isoamylacetate	3-Methylbutyl acetate	123-92-2
Menthol	5-Methyl-2-(propan-2-yl)cyclohexan-1-ol	89-78-1
Vanillin	4-Hydroxy-3-methoxybenzaldehyde	121-33-5

### Cell culture and drug treatment

Porcine aortic endothelial cells were isolated as described [26] and cultured at 37°C, 5% CO_2_ for up to 3 passages in Dulbecco´s modified Eagle´s medium, containing 10% heat-inactivated fetal calf serum (FCS), 100 U/ml penicillin, 0.1 mg/ml streptomycin, and 1.25 μg/ml amphotericin B. Prior to experiments, cells were subcultured into 24-well plates (determination of cGMP formation) or 6-well plates (determination of L-citrulline formation).

### Determination of endothelial cGMP formation

Accumulation of intracellular 3',5'-cyclic GMP (cGMP) was determined as previously described [27]. Briefly, endothelial cells were washed and preincubated with the compounds to be tested for 15 minutes at 37°C in 50 mM Tris buffer, pH 7.4, containing 100 mM NaCl, 5 mM KCl, 1 mM MgCl_2_, 2.5 mM CaCl_2_, 1 mM 3-isobutyl-1-methylxanthine, and 1 μM indomethacin. Reactions were started by addition of the Ca^2+^ ionophore A 23187 (final concentration 1 μM) or the NO donor DEA/NO (final concentration 1 μM) and terminated after 4 minutes by removal of the incubation medium and addition of 0.01 M HCl. Within 1 h, intracellular cGMP was completely released into the supernatant and measured by radioimmunoassay.

### Determination of endothelial L-citrulline formation

NO synthase (NOS) activity in intact cells was determined by monitoring the conversion of L-[^3^H]arginine into L-[^3^H]citrulline as previously described [27]. Briefly, endothelial cells were washed and preincubated with the compounds to be tested for 10 minutes at 37°C in 50 mM Tris buffer, pH 7.4, containing 100 mM NaCl, 5 mM KCl, 1 mM MgCl_2_ and 2.5 mM CaCl_2_. Reactions were started by addition of L-[2,3,4,5-^3^H]arginine (~ 10^6^ dpm) and A 23187 (final concentration 0.3 μM) and terminated after 4 min by washing the cells with chilled incubation buffer. Subsequent to lysis of the cells with 0.01 M HCl, an aliquot was removed for determination of incorporated radioactivity. To the remaining sample, 200 mM sodium acetate buffer (pH 13.0) containing 10 mM L-citrulline was added (final pH ~5.0), and L-[^3^H]citrulline was separated from L-[^3^H]arginine by cation exchange chromatography.

### Determination of soluble guanylate cyclase (sGC) activity

The activity of sGC purified from bovine lung [28] was determined as formation of [^32^P]cGMP from [α-^32^P]GTP. The purified enzyme (50 ng) was incubated at 37°C for 10 min in a final volume of 0.1 ml in the absence or presence of flavorings with the NO donor DEA/NO at concentrations indicated in the figure legends, followed by isolation of [^32^P]cGMP and quantification of radioactivity (Cerenkov radiation) in a β counter.

To test for reversibility of inhibition, sGC (50 ng) was preincubated in the absence or presence of the respective compounds for 5 minutes at 37°C, followed by 50-fold dilution in the assay mixtures and incubation with DEA/NO at 37°C for 10 minutes.

### Determination of cytotoxicity

The viability of endothelial cells was judged by incubation with 1 mM of the flavoring compounds, followed by determination of 3-(4,5-dimethyl-2-thiazolyl)-2,5-diphenyltetrazolium bromide (MTT) to formazan conversion using the Cayman MTT Cell Proliferation Assay Kit purchased through VWR International (Vienna, Austria).

To test for membrane integrity, endothelial cells were assayed for release of lactate dehydrogenase (LDH) into the culture medium. Subsequent to incubation with flavoring compounds (1 mM each) for 1 hour, 20-μl aliquots of the culture media were mixed with 200 μl of reagent buffer (1 mM pyruvate and 0.1 mM NAD^+^ in PBS). The decrease in light absorbance at 340 nm was monitored using a diode array spectrophotometer (Hewlett Packard 8452A). Maximum LDH release was determined by treating the cells with 1% Triton X-100.

### Electrochemical determination of NO

Scavenging of NO by flavoring compounds was studied with a nitric oxide sensor (ISO-NOP, World Precision Instruments, Berlin, Germany), calibrated with acidified nitrite as described previously [29]. Free NO radical was generated by incubation of DEA/NO (1 μM) in 1 ml of 100 mM phosphate buffer, pH 7.4, in the absence or presence of superoxide dismutase (2500 U/ml) at ambient temperature. At the peak concentration of NO, flavoring compounds (10 μl of DSMO stock solutions) were added, DMSO served as vehicle control.

### Determination of cinnamaldehyde in commercial liquids

The concentration of cinnamaldehyde in three commercial liquids was quantified by high-performance liquid chromatography (Agilent 1260 Infinity with diode array detection) on a reversed-phase poroshell 120 EC-C18 column (Agilent Technologies) using 40% methanol and 60% water as mobile phase. Liquids were diluted 1:100 in mobile phase, aliquots of 6 μl were applied onto the column and eluted isocratically with a flow rate of 1.0 ml/min at a column temperature of 25°C. The method was calibrated with authentic cinnamaldehyde (10–300 μM), based on the area of the peak detected at 290 nm (retention time 4.5 min).

### Animals and tissue preparation

Animal experiments were performed in compliance with the Austrian law on experimentation with laboratory animals (last amendment 2013) based on the European Union guidelines for the Care and the Use of Laboratory Animals (European Union Directive 2010/63/EU). Unsexed Sprague-Dawley rats (obtained from Charles River, Sulzfeld, Germany) were housed at the local animal facility in approved cages. They were fed standard chow (Altromin 3023; obtained from Königshofer Futtermittel, Ebergassing, Austria) and received water *ad libitum*. Animals were euthanized in a box that was gradually filled with CO_2_ until vital signs had ceased (cessation of respiration and circulation). Subsequently, the thorax of the animals was opened, the thoracic aorta was removed, cleaned from connective tissue and immediately used for assessment of vessel function.

The killing of animals solely for the use of their organs or tissues is explicitly excluded in Article 3 (page L276/39) of EU Directive 2010/63/EU on protection of animals used for scientific purposes.

### Isometric tension vasomotor studies

For isometric tension measurements, vessel rings with intact endothelium were suspended in 5-ml organ baths containing oxygenated Krebs-Henseleit buffer (concentrations in mM: NaCl 118.4, NaHCO_3_ 25, KCl 4.7, KH_2_PO_4_ 1.2, CaCl_2_ 2.5, MgCl_2_ 1.2, D-Glucose 11; pH 7.4), as previously described in detail [30]. After equilibration for 60 min at the optimal resting tension of 20 mN, maximal contractile activity was determined with a depolarizing solution containing 60 mM K^+^. Rings that did not elicit adequate and stable contraction to high K^+^ were considered damaged and omitted from the study. After washout, tissues were precontracted with the thromboxane mimetic 9,11-dideoxy-9α,11α-epoxy-methanoprostaglandin F_2α_ (U-46619) to an equivalent level (*i*.*e*. ~90% of maximal contraction obtained with high K^+^). When contraction had reached a stable plateau (~after 20 min) cumulative concentration-response curves were established with flavorings. Where indicated, the effects of the flavorings on vascular tone were tested in the presence of the NOS inhibitor N^G^-nitro-L-arginine methyl ester (L-NAME, 0.2 mM).

To examine a possible effect of the tested flavoring compounds on endothelial function and NO bioavailability, cumulative concentration-response curves to acetylcholine (1 nM—10 μM) or DEA/NO (1 nM—10 μM) were performed on U-46619 precontracted rings that had been preincubated with the flavoring compounds for 30 min. The concentrations of the tested flavoring compounds in these experiments represent the highest concentration that did not induce relaxation *per se* (*i*.*e*. 100 μM for eugenol and cinnamaldehyde, and 300 μM for all other compounds).

Effects are expressed as percent of relaxation of U-46619-induced contraction. Concentration-response curves of different ring segments from a single animal were averaged and counted as an individual experiment.

### Statistical analysis

Data are presented as mean values±SEM of n experiments. Where applicable, concentration-response curves were fitted to a Hill-type model giving estimates of agonist potency (EC_50_) and efficacy (E_max_) using Kaleidagraph (Synergy Software, version 4.5). EC_50_ values are reported as geometric means with 95% confidence limits (CI). Analysis of variance (ANOVA) with post hoc Dunnett's test was used for comparison between groups using the Kaleidagraph software. Significance was assumed at p<0.05.

## Results

**Fig 1:**
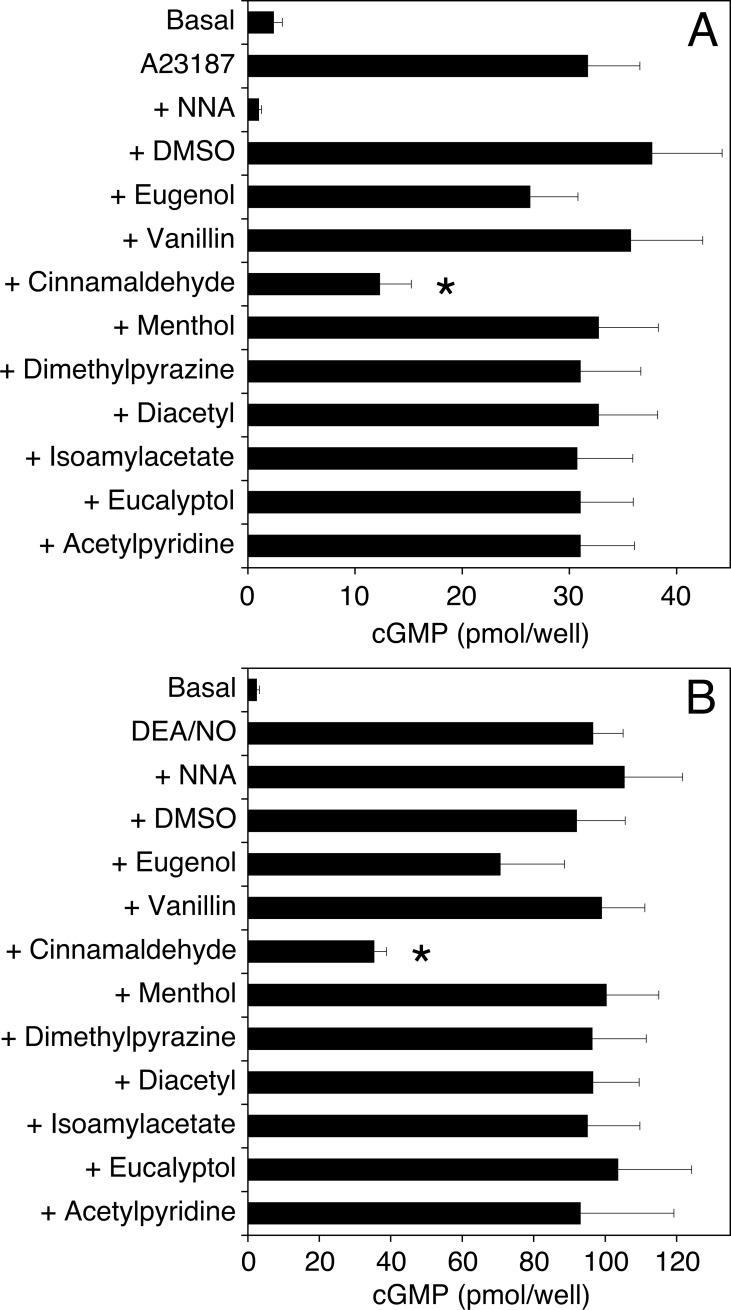
Effect of flavorings on endothelial cGMP formation. Endothelial cells preincubated for 15 min with NNA (0.3 mM), DMSO (0.1%, as solvent control) or the compounds listed (1 mM, each) were stimulated for 4 min with 1 μM A23187 (A) or 1 μM DEA/NO (B), and cGMP formation was determined as described under Materials and Methods. Data are expressed as mean values±SEM (n = 3). *p<0.05 (ANOVA with Dunnett's post hoc test).

**Fig 1** shows the effects of 9 flavoring compounds (1 mM each) on endothelial cGMP accumulation in response to the Ca^2+^ ionophore A23187 (A) and the NO donor DEA/NO (B). The effect of A23187 was completely inhibited by the NOS inhibitor N^G^-nitro-L-arginine (NNA), confirming activation of the endogenous NO/cGMP pathway. Cinnamaldehyde reduced DEA/NO- and A23187-induced cGMP formation to about 35% of controls, while the other compounds had no significant effects.

**Fig 2:**
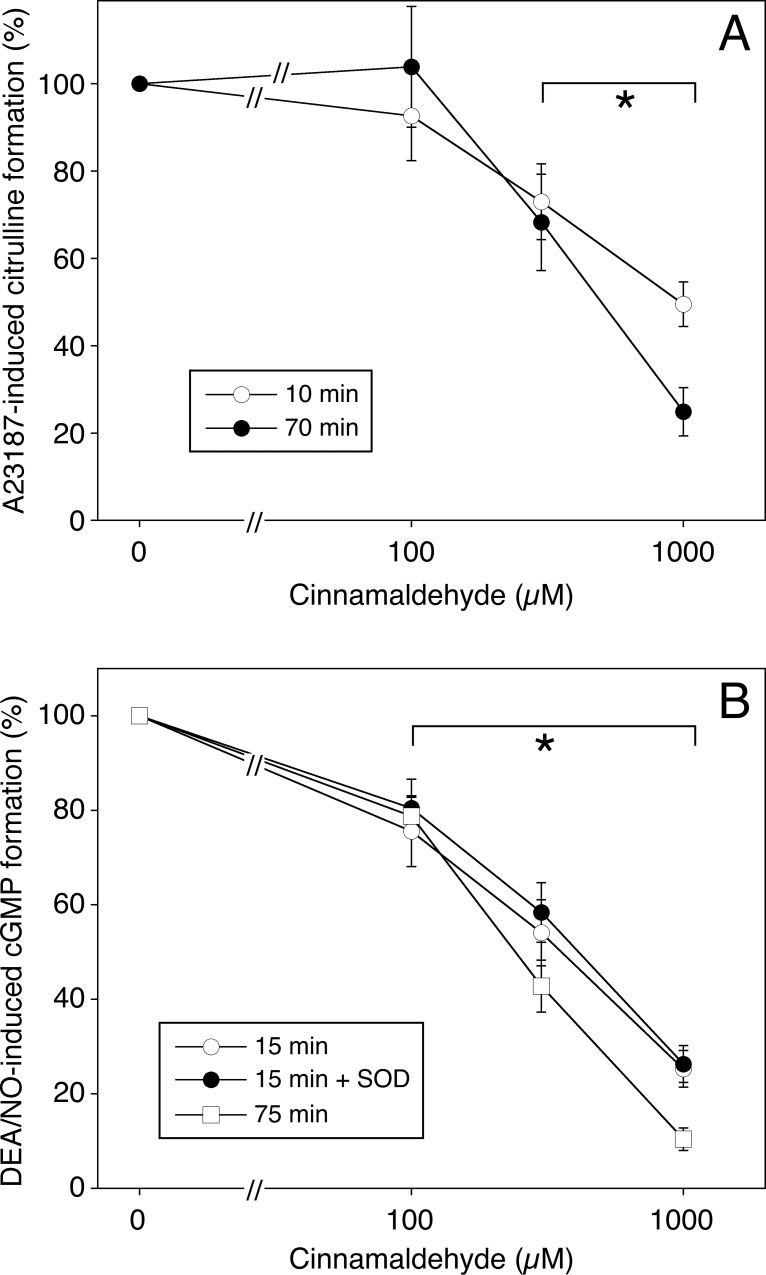
Concentration dependent effect of cinnamaldehyde on endothelial L-citrulline and cGMP formation. A, Endothelial cells were preincubated with increasing concentrations of cinnamaldehyde for 10 min in incubation buffer (open circles) or for 60 min in culture medium and 10 min in incubation buffer (filled circles). Then, cells were stimulated with 1 μM A 23187 (1 μM), and L-citrulline was determined as described under Materials and Methods. Data were normalized to the conversion of L-[^3^H]arginine into L-[^3^H]citrulline measured in non-pretreated cells (= 100%) and are expressed as mean values±SEM (n = 4). B, Endothelial cells were preincubated with increasing concentrations of cinnamaldehyde for 15 min in incubation buffer in the absence (open circles) and presence of 1000 U/ml SOD (filled circles), or for 60 min in culture medium and 15 min in incubation buffer. Then, cells were stimulated with DEA/NO (1 μM) and cGMP formation was determined. Data were normalized to cGMP production measured in non-pretreated cells (= 100%) and are expressed as mean values±SEM (n = 4). *p<0.05 vs. controls in the absence of cinnamaldehyde (ANOVA with Dunnett's post hoc test).

Based on these results we studied the concentration-dependent effects of cinnamaldehyde on A23187-stimulated formation of the endothelial NOS products NO (measured as accumulation of cGMP) and L-citrulline. As shown in **Fig 2A**, endothelial NOS activity, measured as formation of L-citrulline, was significantly inhibited by 0.3 and 1.0 mM cinnamaldehyde, and the effect of the highest concentration was enhanced when the cells were pretreated with the aldehyde for 70 instead of 10 minutes (p<0.05). Direct activation of endothelial sGC with DEA/NO (**Fig 2B**) was more sensitive to cinnamaldehyde with significant inhibition to 25.3±3.9 and 10.4±2.4% of controls upon incubation with 1 mM of the aldehyde for 15 and 75 minutes, respectively. Lack of effect of superoxide dismutase argues against the involvement of superoxide-mediated oxidation of NO.

As shown in **Table 2**, neither of the flavoring compounds, including cinnamaldehyde, exhibited cytotoxicity at up to 1 mM, measured as release of LDH and reduction of MTT after incubation of the cells for 60 minutes.

**Table 2:** Effects of flavorings on endothelial cell viability.

Flavoring	LDH release (% of maximum)	Formazan formation (% of control)
Control	0.4±0.22	100
Acetylpyridine	0.7±0.12	102±4.4
Dimethylpyrazine	0.9±0.12	103±3.7
Eucalyptol	1.0±0.27	99±2.0
Eugenol	0.4±0.23	107±4.6
Isoamylacetate	0.7±0.28	103±3.8
Menthol	0.8±0.03	100±2.9
Vanillin	1.4±0.72	109±6.7
Cinnamaldehyde	1.8±0.50	91±2.6
Diacetyl	0.6±0.03	95±4.6

Values are expressed as % of maximal LDH release induced by treating the cells with 1% Triton or as % of formazan formation measured in untreated cells. Data are mean values±SEM (n = 3, 5 replica each). Data analysis by ANOVA showed that neither of the compounds had significant effects on LDH release (p = 0.11) or formazan formation (p = 0.15).

**Fig 3:**
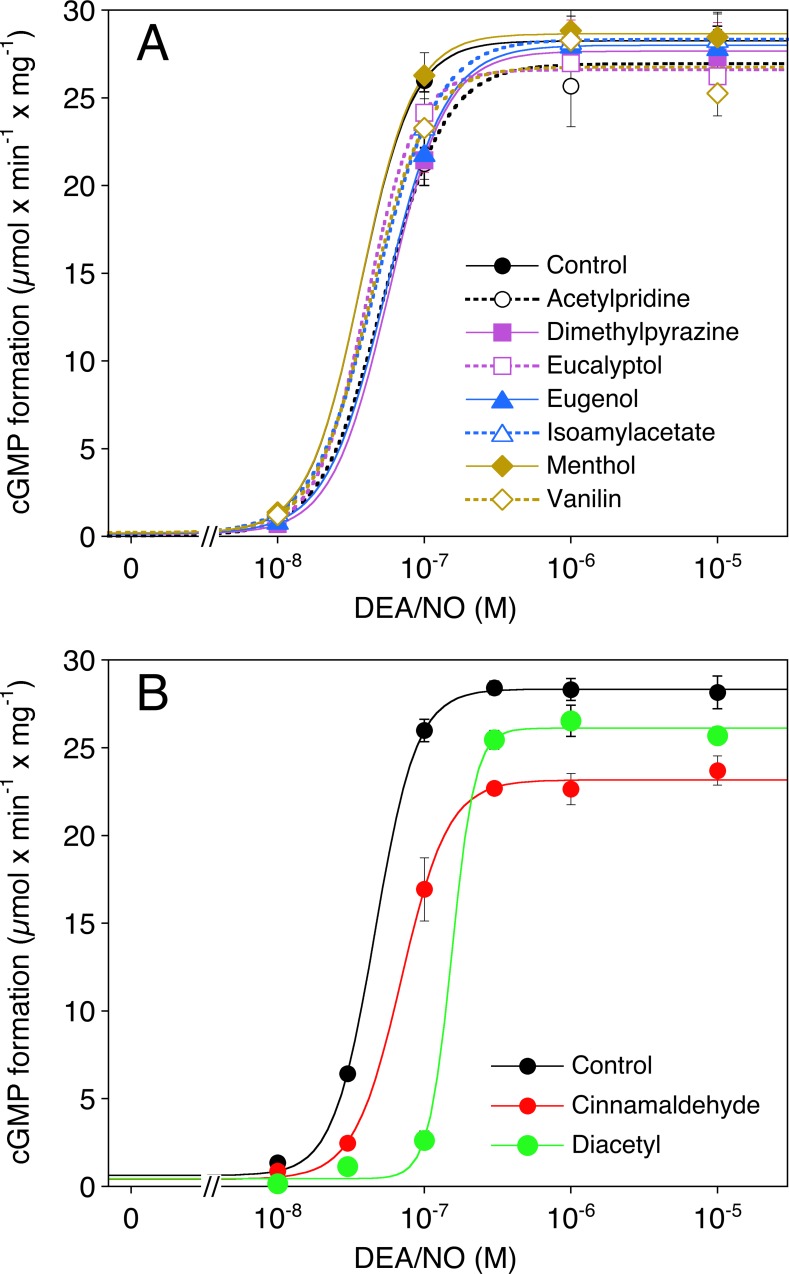
Effect of flavorings on sGC activity. Purified bovine lung sGC (50 ng) was stimulated with increasing concentrations of DEA/NO (0.01–10 μM) in the presence of DMSO (0.1% as solvent control) or flavorings (1 mM each). Formation of cGMP was determined as described in Materials and methods. Data are expressed as mean values±SEM (n = 3). For clarity, the effects of cinnamaldehyde and diacetyl compared to control are shown separately in panel B.

As shown in **Fig 3A**, at a concentration of 1 mM, 7 out of the 9 tested flavorings had no effect on the activity of purified sGC stimulated with increasing concentrations of DEA/NO (0.01–10 μM). Cinnamaldehyde and diacetyl (1 mM each) caused significant rightward shifts of the DEA/NO concentration-response curve from 37 (29–47) to 71 (43–118) and 155 (106–129) nM, respectively (p<0.05). Cinnamaldehyde slightly reduced maximal sGC activity from 28±0.9 to 24±0.8 μmol cGMP x min^-1^ x mg^-1^ (**Fig 3B**), the other flavorings had no significant effects. The E_max_ and EC_50_ values obtained with the various compounds are listed in S1 **Table**.

**Fig 4:**
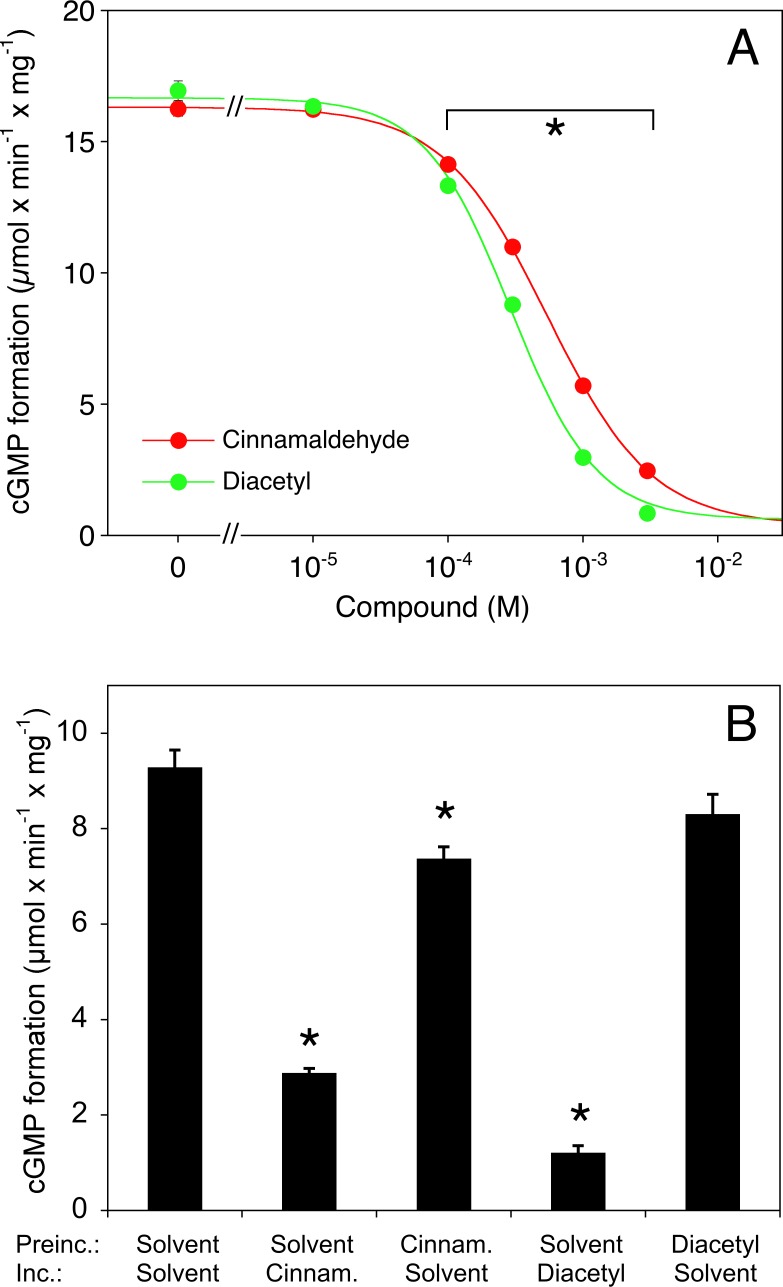
**Effects of cinnamaldehyde and diacetyl concentration on sGC activity (A) and reversibility of inhibition (B).** A, Purified bovine lung sGC (50 ng) was stimulated with DEA/NO (0.05 μM) in the presence of DMSO (0.1% as solvent control) or increasing concentrations of cinnamaldehyde and diacetyl (0.01–3 mM each). B, The enzyme (50 ng) was pre-incubated with DMSO (0.1% as solvent control) or flavorings (1 mM each) for 5 minutes and subsequently diluted 50-fold in reaction mixture, followed by determination for enzyme activity by incubation for 10 minutes in the presence of 0.05 μM DEA/NO in the absence or presence of the flavorings (1 mM each). Data are expressed as mean values±SEM (n = 3). Note that the error bars in panel A are smaller than the symbols. *p<0.05 vs. controls in the absence of cinnamaldehyde (ANOVA with Dunnett's post hoc test).

The effects of cinnamaldehyde and diacetyl on DEA/NO-stimulated sGC were concentration-dependent with IC_50_ values of 0.56 (0.54–0.58) and 0.29 (0.24-.36) mM, respectively (**Fig 4A**) and largely (cinnamaldehyde) or completely (diacetyl, p<0.05) reversed by 50-fold dilution of the enzyme that had been pre-incubated for 5 minutes in the presence of 1 mM of these compounds (**Fig 4B**).

**Fig 5:**
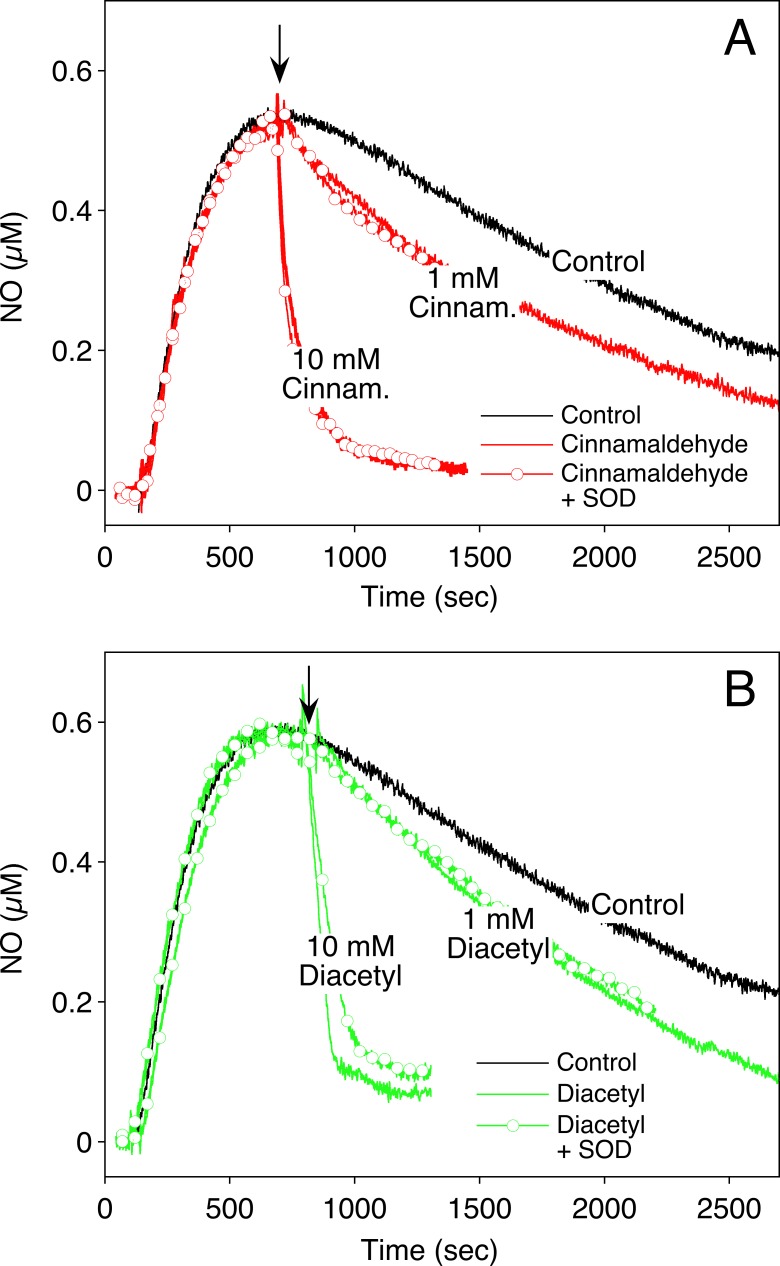
**Inactivation of NO by cinnamaldehyde (A) and diacetyl (B).** NO formation was induced by adding DEA/NO (final concentration 1 μM) to 100 mM phosphate buffer, pH 7.4. At time points indicated by arrows, cinnamaldehyde or diacetyl was added. Experiments performed in the presence of SOD (2500 U/ml) are marked with open circles. Original traces each representative for 3 experiments are shown.

The data with purified sGC indicated that high concentrations of cinnamaldehyde and diacetyl may scavenge NO. This was confirmed by measuring the concentration-time profile of NO released from DEA/NO in the absence and presence of the flavoring compounds with a NO-sensitive electrode. As shown in **Fig 5**, at 10 mM both compounds led to a pronounced decrease of the recorded NO signals, while only moderate effects were observed at 1 mM. Thus, we cannot exclude that direct effects on sGC contribute to reduced cGMP formation in the presence of the flavorings. Scavenging of NO was not prevented by superoxide dismutase, indicating that it was due to a direct reaction with NO rather than generation of superoxide anions and peroxynitrite formation.

**Fig 6:**
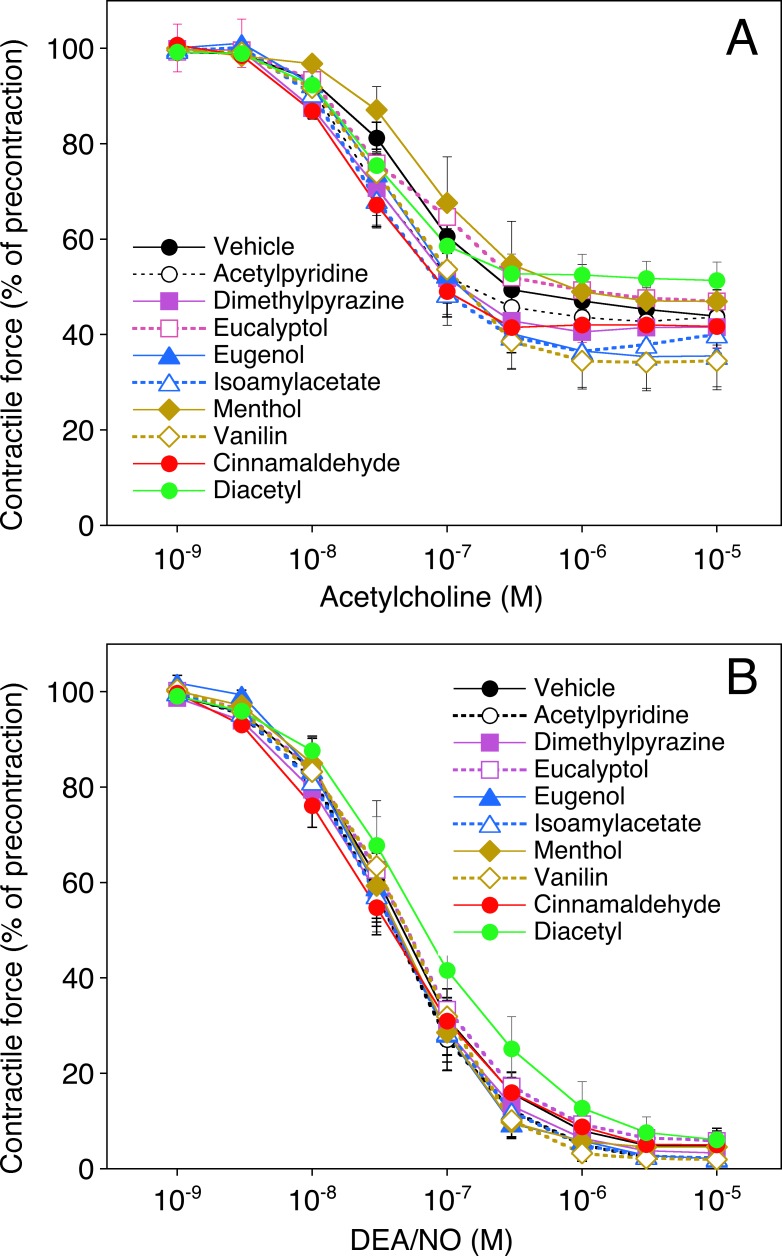
**Effects of flavorings on relaxation of aortic rings to acetylcholine (A) or DEA/NO (B).** The concentration-response to acetylcholine (1 nM—10 μM) or DEA/NO (1 nM—10 μM) was measured with U-46619 pre-contracted rings that had been preincubated with the flavoring compounds for 30 min. The compounds were applied at the highest concentrations that did not induce relaxation on their own (0.1 mM for eugenol and cinnamaldehyde, 0.3 mM for all other compounds; see Fig 7). Concentration-response curves obtained with different ring segments from a single animal were averaged and counted as an individual experiment. Data are expressed as mean values±SEM obtained in 6 (vehicle), 5 (cinnamaldehyde, diacetyl) or 3 experiments (all other flavorings).

The effects of the flavoring compounds on blood vessel function were tested in organ bath experiments with isolated rat aortic rings. As shown in **Fig 6**, the compounds (0.1–0.3 mM) had no significant effects (p>0.05, ANOVA) on vascular relaxation to acetylcholine (A) or DEA/NO (B) that occurred with EC_50_ values of 55 (32–94) nM and 43 (31–61) nM, respectively. The E_max_ and EC_50_ values obtained with the various compounds are given in S2 **Table**.

**Fig 7:**
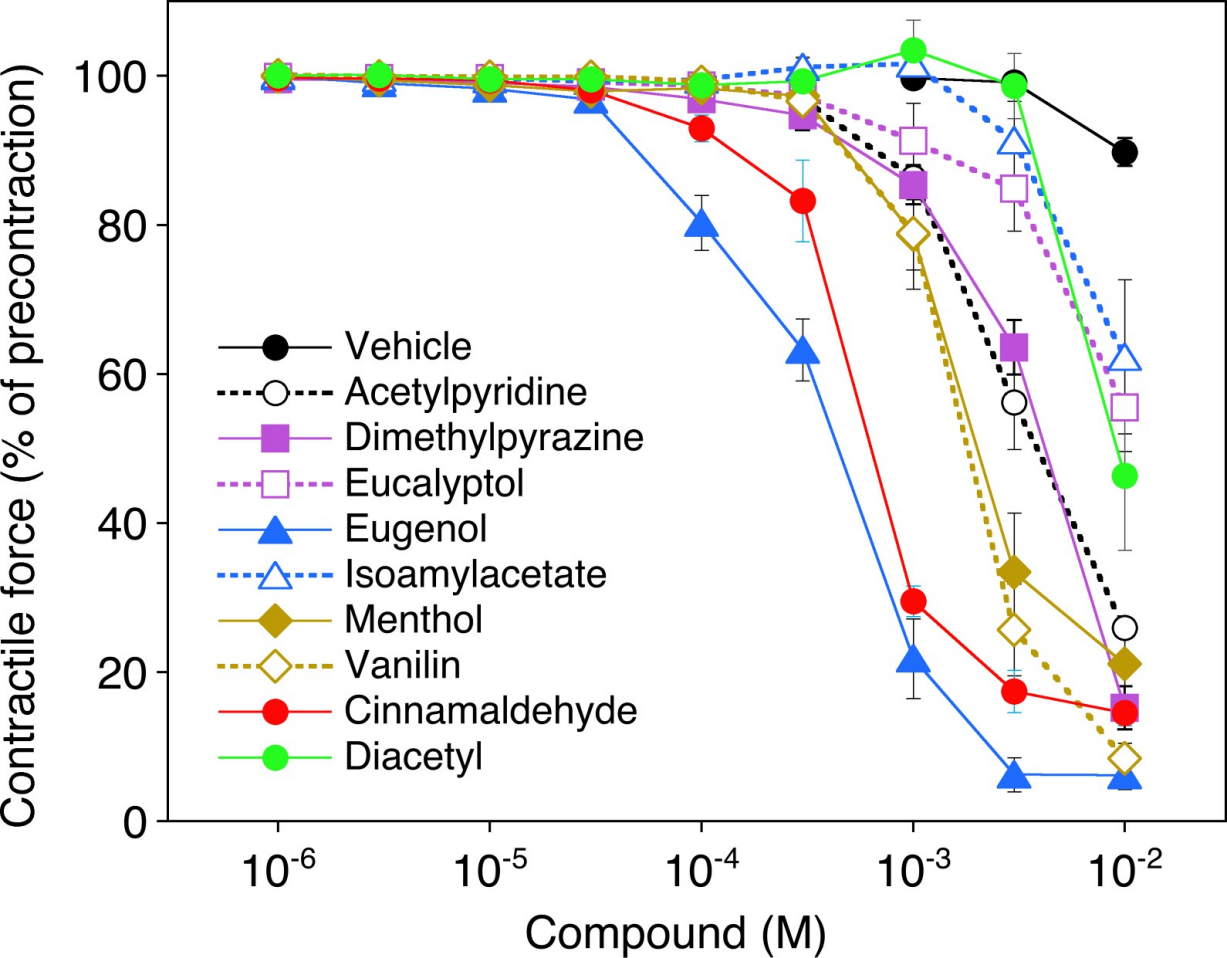
Effects of flavorings on vascular tone. The concentration-response to the various compounds (1 μM—10 mM) or vehicle (DMSO) was measured with U-46619 precontracted aortic rings. Concentration-response curves obtained with different ring segments from a single animal were averaged and counted as an individual experiment. Data are expressed as mean values±SEM (n = 6).

**Fig 7** shows that most of the flavorings caused relaxation of pre-contracted vessels, with EC_50_ values ranging from about 0.5 mM [eugenol 0.4 (0.3–0.6) mM and cinnamaldehyde 0.5 (0.4–0.7) mM] to 1.7–4.2 mM (vanillin, menthol, acetylpyridine, and 2,5-dimethylpyrazine). Diacetyl, isoamyl acetate and eucalyptol had no effects below 10 mM. Neither of these effects were blocked by the NOS inhibitor L-NAME (0.2 mM; see S3 **Fig**, indicating that the effects of the flavorings on vascular smooth muscle tone are not mediated by the endothelial NO synthase/cGMP pathway. The EC_50_ values obtained with the various compounds in the absence and presence of L-NAME are given in S3 **Table**.

To elucidate the potential mechanisms of relaxation induced by cinnamaldehyde and eugenol we studied their effects on accumulation of cAMP and cGMP in endothelium-denuded aortic rings. As shown in S4 **Table**, neither of the two compounds had significant effects on cyclic nucleotide levels at up to 10 mM. However, similarly to verapamil, both flavorings significantly inhibited CaCl_2_-induced contraction of rat aortas (S2 **Fig**), indicating interference with Ca^2+^ entry into vascular smooth muscle cells.

Since Cinnamaldehyde was shown to slowly decompose to yield benzaldehyde upon heating [31], we tested the potency of benzaldehyde to relax aortic rings. As shown in S4 **Fig**, benzaldehyde exhibited similar potency as the parent compound, suggesting a common mechanism of action and excluding pronounced vascular effects of thermal degradation of cinnamaldehyde.

Three commercially available liquids were purchased and analyzed for cinnamaldehyde content by HPLC. As shown in **Table 3**, the three liquids contained 1.5 to 6.9 mM cinnamaldehyde, corresponding to 0.2–0.91 mg/ml. The measured values were strikingly different from the information provided by the manufacturer on the respective data sheets (see **Table 3**). After independent triple checking of our results, the discrepancy was communicated to the manufacturer but has remained unresolved. Most likely the values provided on the data sheets refer to the concentrated flavors added to nicotine base and not to the cinnamaldehyde concentrations of the finished liquids.

**Table 3:** Cinnamaldehyde content in liquids.

	Cinnamaldehyde		
	measured		according to happy liquid
Liquids	(mg/ml)	(mM)	(mg/ml)
Auszogne mit Zimt	0.56±0.002	4.24±0.017	3
Glühwein	0.91±0.006	6.89±0.044	10–20
Apfelstrudel	0.20±0.002	1.53±0.019	not specified

Measured values are presented as mean±SEM (n = 3)

## Discussion

The present results indicate that NO scavenging does not necessarily reflect endothelial dysfunction and impaired vascular tone, as claimed in a previous study [25]. Among the flavoring compounds tested, only cinnamaldehyde and diacetyl reduced NO bioavailability, evident as reduced activity of NO-stimulated purified sGC and scavenging of the NO signal recorded with a NO-sensitive electrode. Lack of effect of superoxide dismutase argues against superoxide-mediated NO inactivation and suggests a direct reaction of the compounds with free NO radical. Our observation that the effects of both compounds were fully reversed upon dilution of the reaction mixtures after pre-incubation of the enzyme with cinnamaldehyde or diacetyl (see **Fig 4B**) indicate that sGC is not modified by these compounds in an irreversible manner.

Fetterman *et al*. [25] reported significant NO scavenging by vanillin, menthol, cinnamaldehyde, and acetylpyridine. Except for cinnamaldehyde neither of these compounds had any effects on the concentration-dependent stimulation of sGC, the most sensitive physiological target of NO. The discrepancy may be related to the use of diaminofluorescein (DAF) fluorescence as an indicator for NO formation in the previous study, a method which can result in artifacts [32–34] due to reactions of the dye with the test compounds.

Endothelium-derived NO is an important modulator of blood vessel function. Damage of the vascular endothelium, resulting in the impaired release of NO and other vasoactive agents, may contribute to hypertension, thrombotic events, and inflammation [35, 36]. However, in some cases direct effects on vascular smooth muscle prevail over reduced NO bioavailability as illustrated by the pronounced relaxation of rat aortic rings to cinnamaldehyde, which showed NO scavenging at similar concentrations. It has been shown that cinnamaldehyde exhibits a wide variety of beneficial effects, including improved glucose handling in type-II diabetes [37], protection against M. Alzheimer [38], antibacterial activity [39] and general anti-oxidant effects that confer protection against natural and artificial toxicants [40]. In addition, it is well established that cinnamaldehyde causes endothelium-independent vasodilation [41–43]. However, the role of the endothelium is controversial, as it was reported that activation of endothelial transient receptor potential ankyrin-1 (TRPA1) contributes to relaxation [44]. Since cinnamaldehyde did not increase endothelial L-citrulline formation in the absence of A23187 (see S1 **Fig**) but inhibited Ca^2+^ activation of NOS at higher concentrations, TRPA1-mediated activation of endothelial NO synthesis appears to play no role or may be masked by other processes in porcine aortic endothelial cells. Given the low potency of the aldehyde, we did not further investigate this effect.

Cinnamaldehyde has a high margin of safety and is generally recognized as safe (GRAS) by the Food and Drug Administration. We observed no toxicity by up to 1 mM cinnamaldehyde on endothelial cells, judged by LDH release and MTT reduction. Similarly, Farsalinos *et al*. found only moderate toxicity of undiluted cinnamon liquid on a cardiomyoblast cell line [45]. However, it has been shown that cinnamaldehyde containing vapor is toxic to lung epithelial cells [24], indicating that inhalation of aerosols containing high concentrations of cinnamaldehyde, as reported recently [46], could be harmful to the lungs.

With an EC_50_ of about 0.5 mM, eugenol was the most potent vasorelaxant among the tested compounds. Eugenol is a natural ingredient of a wide variety of plants, including beans and coffee, and is used in the food industry as preservative and flavoring agent. Eugenol exhibits pronounced anti-inflammatory and anti-oxidant activities (for review see Barboza *et al*. [47]). Several previous studies have demonstrated vascular relaxation and reduction of blood pressure by eugenol and eugenol containing essential oils [48–52]. There is general agreement that eugenol acts mainly *via* inhibition of Ca^2+^ influx, but some studies reported on partial inhibition of vasodilation by NOS inhibitors, indicating that endothelium-derived NO might contribute to relaxation [48, 51]. However, in agreement with other studies [50, 52], we observed no effect of the NOS inhibitor L-NAME on eugenol-induced relaxation (see S3 **Fig**), and at 1 mM neither eugenol nor cinnamaldehyde increased NOS-catalyzed L-citrulline formation in cultured endothelial cells (see S1 **Fig**) but blocked Ca^2+^-induced contraction of endothelium-denuded blood vessels (see S2 **Fig**). The reason for the conflicting results is unknown.

Vanillin, menthol, acetylpyridine, and 2,5-dimethylpyrazine caused vasorelaxation with EC_50_ values in the range of 2 to 5 mM. Inhibition of Ca^2+^ influx appears to mediate vasodilation to vanillin [53] and menthol [54–56], the vascular effects of acetylpyridine and 2,5-dimethylpyrazine have not been reported so far. Relaxation observed with 10 mM isoamyl acetate and eucalyptol is probably unspecific and considered as irrelevant.

Previous *in vitro* studies indicate that some refill liquids of e-cigs may be cytotoxic to cultured endothelial cells, but the role of flavors has not been addressed [57–59]. Of note, since harmful effects on blood vessels would occur independently of the route of administration, toxicity to endothelial cells would question the safety of compounds used for decades as food flavors. The present results do not support the claim of Fetterman *et al*. [25] that flavoring compounds used in refill liquids of e-cigs impair blood vessel function. If having any *in vivo* effects at all, some of these compounds may be vasoprotective by reducing vascular tone and, according to previous studies, inhibition of inflammatory processes. To judge the biological relevance of the observed effects we estimated the plasma concentration that might occur upon inhalation of aerosols containing cinnamaldehyde, a compound with a known pharmacokinetic profile in rats. The highest concentration of this compound measured in three selected, commercially available cinnamon-related liquids was 6.9 mM. Assuming a plasma volume of 3 liters, 100% bioavailability and lack of distribution, metabolism or clearance of cinnamaldehyde, consumption of 10 ml of liquid (69 μmol) would result in a plasma concentration of 23 μM, a value that is below the range of active concentrations in our experiments. Human pharmacokinetic data are not available, but according to a study with rats [60], these assumptions are highly unrealistic and the expected plasma concentrations will be much lower. Upon intravenous application, cinnamaldehyde was rapidly and efficiently metabolized to cinnamic acid, which was unstable itself and exhibited a biological half-life of about 7 minutes. Intravenous bolus application of 25 mg/kg (corresponding to 1.5 g or 11.4 mmol for a human with 60 kg body weight) resulted in a maximal plasma concentration of 20 μM [60]. In a more recent study, oral administration of 500 mg/kg to rats (corresponding to 30 g or 228 mmol for a human with 60 kg body weight), resulted in a maximal plasma concentration of about 2 μM cinnamaldehyde [61]. Since consumption of flavoring agents by vaping is distributed over several hours, the effective plasma concentration is expected to remain in the nanomolar range even upon heavy vaping. Similar considerations may apply to eugenol and the other, even less potent compounds tested.

## Limitations of the study

The present results obtained *in vitro* with purified sGC, cultured endothelial cells and isolated blood vessels do not necessarily reflect how vascular NO signaling is affected by the flavoring agents *in vivo*. It is conceivable that interaction of the compounds with NO/cGMP signaling in vascular smooth muscle cells differs from the effects observed in endothelial cells.

In addition, vaporization of liquids in e-cigs may cause thermal degradation of some compounds, resulting in formation of toxic decomposition products. Although the data shown in S4 **Fig** argue against a considerable effect of cinnamaldehyde decomposition to benzaldehyde, we cannot exclude harmful effects of unknown degradation products that were not studied.

Finally, it should be mentioned that we tested for single, acute effects and did not consider potential consequences of chronic inhalation. Besides the tested flavorings, relatively large amounts of propylene glycol and glycerol are inhaled by users. Although inhalation of these agents is generally considered as safe, longterm adverse effects cannot be excluded.

## Conclusions

Our data indicate that flavorings typically present in e-cig refill liquids do not cause endothelial dysfunction that would result in impaired vasodilation upon acute exposure. In contrast, most of the tested compounds caused endothelium-independent vasorelaxation, albeit at fairly high concentrations that appear to exceed by far the plasma concentrations expected to occur upon vaping flavored liquids.

## Supporting information

S1 Table E_max_ and EC_50_ values calculated from the data shown in Fig 3. (PDF)

S2 Table E_max_ and EC_50_ values calculated from the data shown in Fig 6. (PDF)

S3 Table EC_50_ values calculated from data shown in Fig 7 and S3 Fig. (PDF)

S4 Table Effects of cinnamaldehyde and eugenol on cAMP and cGMP levels in endothelium-denuded rat aortic rings. (PDF)

S1 Fig Lack of direct effects of eugenol and cinnamaldehyde on endothelial L-citrulline formation. (PDF)

S2 Fig Effects of cinnamaldehyde and eugenol on CaCl2-induced contractile response in endothelium-denuded rat aortic rings. (PDF)

S3 Fig Lack of effect of the NOS inhibitor NG-nitro-L-arginine methyl ester (L NAME) on vasodilation induced by flavorings. (PDF)

S4 Fig Effect of benzaldehyde, the major product of thermal degradation of cinnamaldehyde on relaxation of aortic rings to acetylcholine (A), DEA/NO (B) or vascular tone (C).
(PDF)
