# Supplementary material for: Effects of flavoring compounds used in electronic cigarette refill liquids on endothelial and vascular function
Source: PLoS One. 2019 Sep 9;14(9):e0222152. doi: 10.1371/journal.pone.0222152 (PMC6733504; doi:10.1371/journal.pone.0222152)
Supplement: S1 Table — (PDF) [file pone.0222152.s001.pdf]

S1 Table. E_{max} and EC_{50} values calculated from the data shown in Fig. 3

Flavoring	E_{max} (μmol cGMP x min^{-1} x mg^{-1})	E_{C50} (nM)
Control	28±0.9	37 (29-47)
Acetylpyridine	28±0.8	53 (26-107)
Dimethylpyrazine	27±0.7	59 (37-94)
Eucalyptol	26±0.9	41 (30-56)
Eugenol	28±1.1	57 (27-121)
Isoamylacetate	28±0.7	45 (18-112)
Menthol	28±0.4	37 (28-48)
Vanillin	25±0.6	43 (33-55)
Cinnamaldehyde	24±0.8*	71(43-118)*
Diacetyl	26±0.5	155 (106-229)*

Data shown are mean values±SEM (E_{max}) or mean values with 95% confidence interval (EC_{50}) from three experiments. *$p<0.05$ vs. control as determined by ANOVA and Dunnett's post hoc test.
