# Supplementary material for: Effects of flavoring compounds used in electronic cigarette refill liquids on endothelial and vascular function
Source: PLoS One. 2019 Sep 9;14(9):e0222152. doi: 10.1371/journal.pone.0222152 (PMC6733504; doi:10.1371/journal.pone.0222152)
Supplement: S2 Table — (PDF) [file pone.0222152.s002.pdf]

S2 Table. E_{max} and EC_{50} values calculated from the data shown in Fig. 6

Flavoring	Acetylcholine		DEA/NO	
	E_{max} (%)	EC_{50} (nM)	E_{max} (%)	EC_{50} (nM)
Vehicle	41±2.5	55 (32-94)	4±0.8	43 (31-61)
Acetylpyridine	43±6.6	31 (13-74)	2±1.1	18 (15-93)
Dimethylpyrazine	41±5.7	32 (8-131)	2±1.0	39 (17-90)
Eucalyptol	47±2.4	39 (16-95)	6±2.6	42 (15-118)
Eugenol	36±2.0	43 (9-205)	3±2.2	36 (8-161)
Isoamylacetate	36±7.7	30 (12-73)	2±0.9	37 (22-67)
Menthol	46±2.3	75 (13-449)	4±3.1	38 (17-85)
Vanilin	34±5.7	46 (10-222)	2±0.6	43 (5-364)
Cinnamaldehyde	41±3.6	35 (22-54)	4±1.1	33 (15-71)
Diacetyl	50±3.4	30 (20-44)	4±1.1	65 (27-159)

Data shown are mean values±SEM (E_{max}) or mean values with 95% confidence interval (EC_{50}) from 6 experiments (vehicle), 5 experiments (cinnamaldehyde, diacetyl) or 3 experiments (all other flavorings). Data analysis by ANOVA showed that none of the values obtained with flavorings were significantly different from vehicle controls ($p>0.05$).
