# Supplementary material for: Effects of flavoring compounds used in electronic cigarette refill liquids on endothelial and vascular function
Source: PLoS One. 2019 Sep 9;14(9):e0222152. doi: 10.1371/journal.pone.0222152 (PMC6733504; doi:10.1371/journal.pone.0222152)
Supplement: S3 Table — (PDF) [file pone.0222152.s003.pdf]

S3 Table. EC$_{50}$ values calculated from data shown in Fig. 7 and Fig. S3

Flavoring	Control EC$_{50}$ (mM)	L-NAME EC$_{50}$ (mM)
Vehicle	n.d.	n.d.
Acetylpyridine	3.7 (2.2-6.3)	5.6 (2.9-10.4)
Dimethylpyrazine	4.2 (3.4-5.1)	5.2 (4.0-6.8)
Eucalyptol	n.d.	n.d.
Eugenol	0.4 (0.3-0.6)	0.5 (0.2-1.1)
Isoamylacetate	n.d.	n.d.
Menthol	1.6 (1.2-2.2)	1.4 (1.1-1.8)
Vanilin	1.7 (1.1-2.6)	1.8 (1.2-2.9)
Cinnamaldehyde	0.5 (0.4-0.7)	0.6 (0.4-0.7)
Diacetyl	n.d.	n.d.

Data shown are mean values with 95% confidence interval from 6 experiments. Data analysis by ANOVA showed that L-NAME had no significant effect on relaxation induced by any of the tested compounds ($p>0.05$). n.d., not determinable.
