# Supplementary material for: Effects of flavoring compounds used in electronic cigarette refill liquids on endothelial and vascular function
Source: PLoS One. 2019 Sep 9;14(9):e0222152. doi: 10.1371/journal.pone.0222152 (PMC6733504; doi:10.1371/journal.pone.0222152)
Supplement: S4 Table — (PDF) [file pone.0222152.s004.pdf]

S4 Table. Effects of cinnamaldehyde and eugenol on cAMP and cGMP levels in endothelium-denuded rat aortic rings.

Flavoring	cAMP (fmol x mg^{-1})	cGMP (fmol x mg^{-1})
Basal	178±24	91±9
Cinnamaldehyde (1 mM)	200±36	127±10
Cinnamaldehyde (10 mM)	161±7	86±7
Eugenol (1 mM)	162±9	81±4
Eugenol (10 mM)	171±24	71±18
Forskolin (10 μM)	9309±488 *	n.d.
DEA/NO (10 μM)	n.d.	4917±1483 *

Endothelium-denuded rings, treated with test compounds as described in the Materials and methods section of the main text, were freeze-clamped when maximal relaxation had been reached. Rings were homogenized in 5% trichloroacetic acid and analyzed for cAMP and cGMP by radioimmunoassay. Forskolin and DEA/NO were used to induce maximal accumulation of cAMP and cGMP, respectively. Data shown are mean values±SEM of 3 (forskolin and DEA/NO) or 6 (basal, cinnamaldehyde and eugenol) rings; n.d., not determined, *$p<0.05$ vs. basal (ANOVA and Dunnett's post hoc test).
