# Supplementary material for: Effects of flavoring compounds used in electronic cigarette refill liquids on endothelial and vascular function
Source: PLoS One. 2019 Sep 9;14(9):e0222152. doi: 10.1371/journal.pone.0222152 (PMC6733504; doi:10.1371/journal.pone.0222152)
Supplement: S1 Fig — (PDF) [file pone.0222152.s005.pdf]

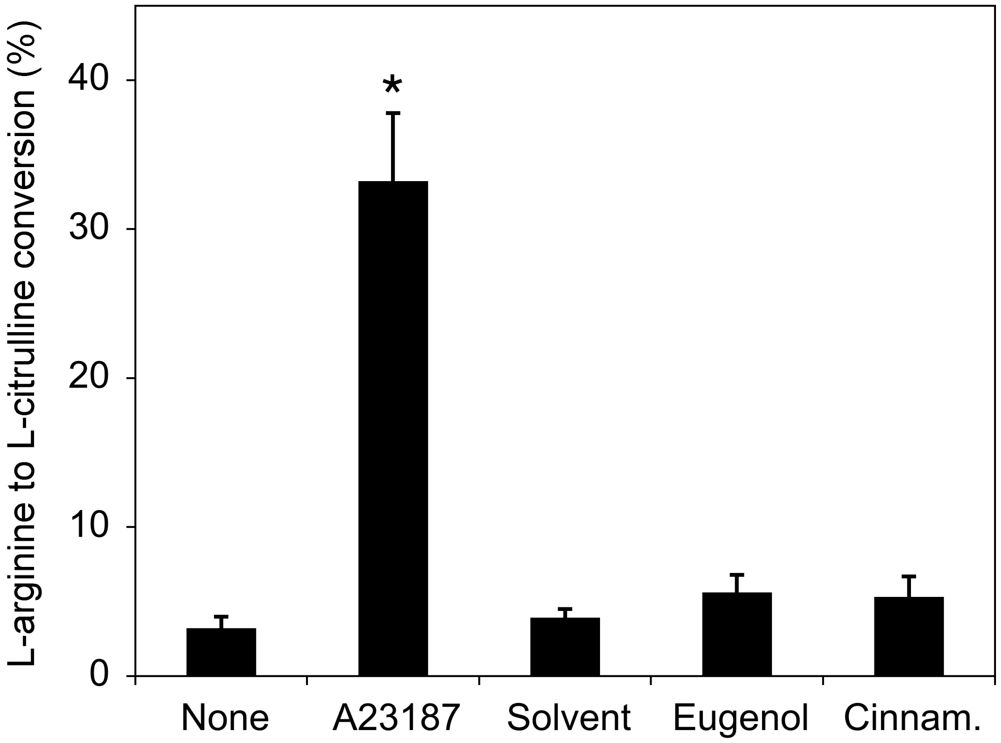

S1 Fig. **Lack of direct effects of eugenol and cinnamaldehyde on endothelial L-citrulline formation.** Endothelial cells were incubated for 10 min with 1 µM A23187, 1 mM eugenol, 1 mM cinnamaldehyde or 0.1% DMSO (solvent). Conversion of L-[^{3}H]arginine into L-[^{3}H]citrulline was determined as described in the Materials and methods section of the main text. Data are mean values±SEM (n=3). *$p<0.05$ vs. control as determined by ANOVA and Dunnett's post hoc test.
