# Supplementary material for: Effects of flavoring compounds used in electronic cigarette refill liquids on endothelial and vascular function
Source: PLoS One. 2019 Sep 9;14(9):e0222152. doi: 10.1371/journal.pone.0222152 (PMC6733504; doi:10.1371/journal.pone.0222152)
Supplement: S2 Fig — (PDF) [file pone.0222152.s006.pdf]

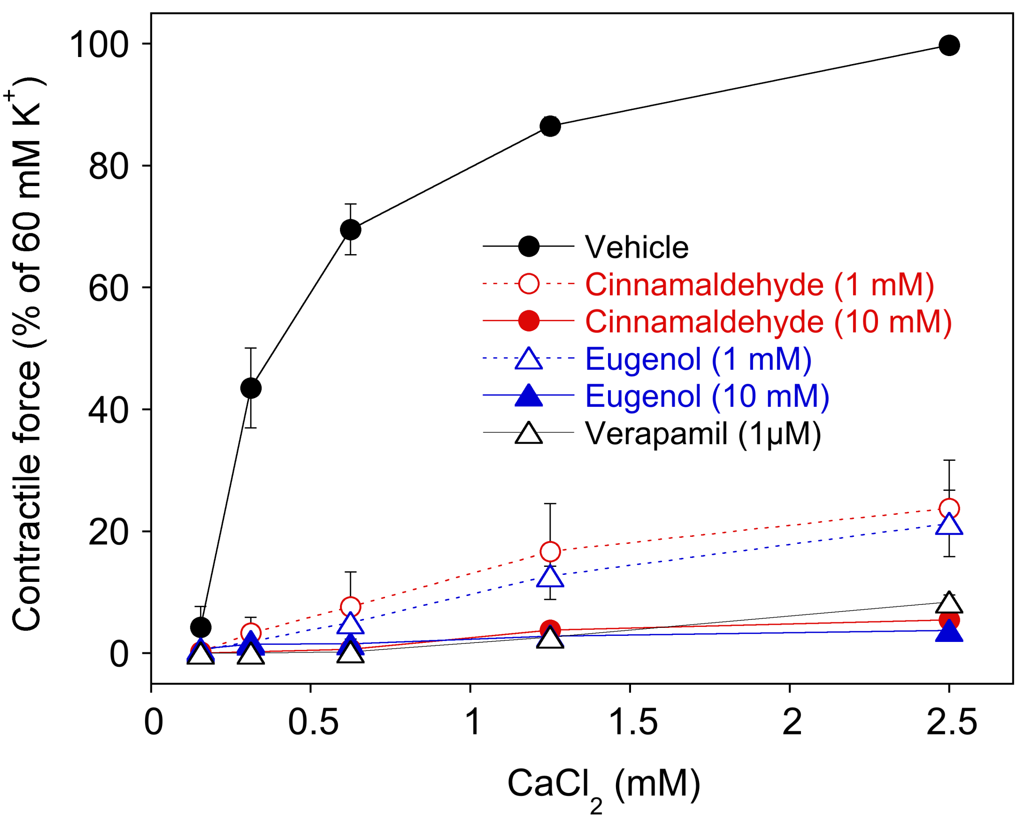

S2 Fig. Effects of cinnamaldehyde and eugenol on $CaCl_2$-induced contractile response in endothelium-denuded rat aortic rings. Rings were treated with Ca^{2+}-free high K^+ solution (containing 0.1 mM EGTA and 60 mM K^+). The Ca^{2+}-free preparations were then cumulatively contracted with $CaCl_2$ (0.156-2.5 mM) in the absence or presence of cinnamaldehyde or eugenol. The established L-type Ca^{2+} channel blocker verapamil was used as reference compound. Data were normalized to contraction induced by 60 mM K^+ in the presence of 2.5 mM $CaCl_2$ (=100%). Concentration-response curves obtained with different ring segments from a single animal were averaged and counted as an individual experiment. Data are expressed as mean values±SEM (n=3). All compounds significantly inhibited $CaCl_2$-induced contraction ($p<0.05$ determined by ANOVA and Dunnett's post hoc test).
