# Supplementary material for: Effects of flavoring compounds used in electronic cigarette refill liquids on endothelial and vascular function
Source: PLoS One. 2019 Sep 9;14(9):e0222152. doi: 10.1371/journal.pone.0222152 (PMC6733504; doi:10.1371/journal.pone.0222152)
Supplement: S3 Fig — (PDF) [file pone.0222152.s007.pdf]

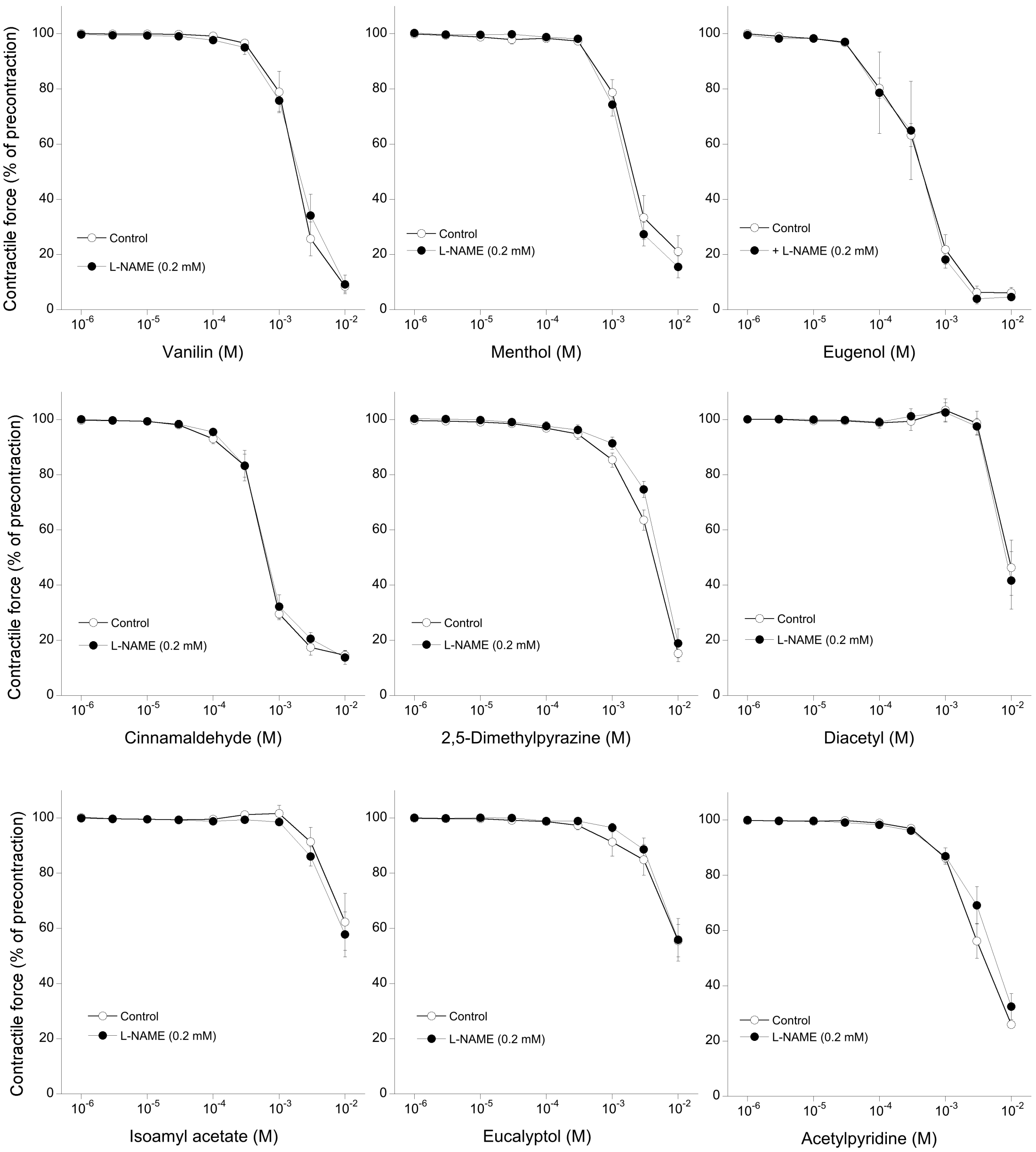

S3 Fig. Lack of effect of the NOS inhibitor N^G-nitro-L-arginine methyl ester (L-NAME) on vasodilation induced by flavorings. Concentration-response curves obtained with different ring segments from a single animal were averaged and counted as an individual experiment. Data are expressed as mean values±SEM (n=6). Statistical analysis of the data is shown in Table S3.
