# Supplementary material for: Effects of flavoring compounds used in electronic cigarette refill liquids on endothelial and vascular function
Source: PLoS One. 2019 Sep 9;14(9):e0222152. doi: 10.1371/journal.pone.0222152 (PMC6733504; doi:10.1371/journal.pone.0222152)
Supplement: S4 Fig — Effect of benzaldehyde, the major product of thermal degradation of cinnamaldehyde on relaxation of aortic rings to acetylcholine (A), DEA/NO (B) or vascular tone (C). (PDF) [file pone.0222152.s008.pdf]

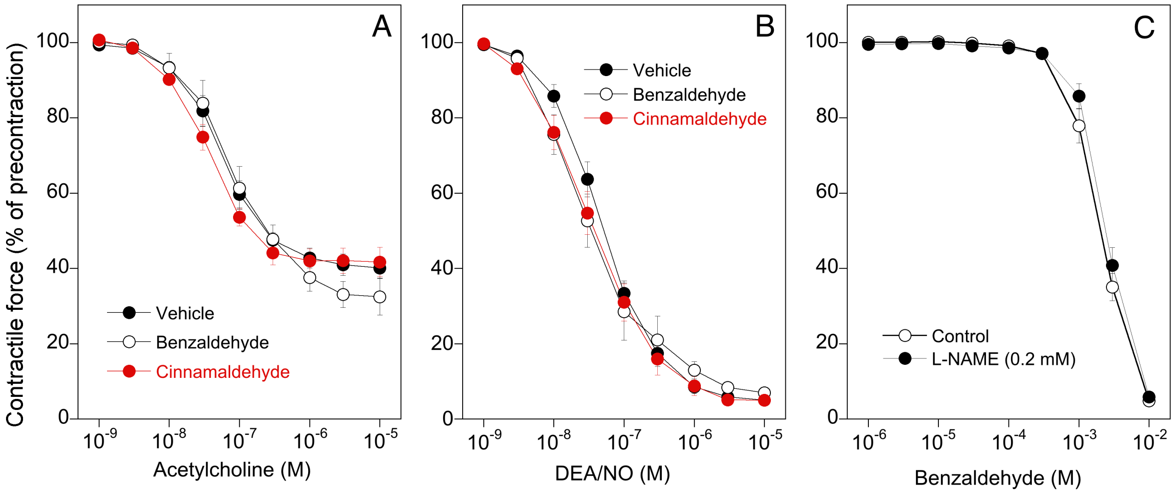

S4 Fig. Effect of benzaldehyde, the major product of thermal degradation of cinnamaldehyde on relaxation of aortic rings to acetylcholine (A), DEA/NO (B) or vascular tone (C). Benzaldehyde (300 μM) neither affected acetylcholine (EC_{50}: 68 (11-439) nM; E_{max}: 32±5.5%) nor DEA/NO (EC_{50}: 28 (6-125) nM, E_{max}: 6±3.2%) - induced relaxation. Benzaldehyde relaxed precontracted vessels in the absence or presence of L-NAME with EC_{50} values of 2.2 (1.5-3.0) and 2.5 (1.9-3.3) mM, respectively (p=0.45, ANOVA). The vehicle and cinnamaldehyde data from Fig. 7 are shown for comparison. Concentration-response curves obtained with different ring segments from a single animal were averaged and counted as an individual experiment. Data are expressed as mean values±SEM (n=3 (A, B) and 6 (C)).
